# Digoxin Dilemma: Diagnosing Toxicity Amidst Dementia

**DOI:** 10.19102/icrm.2025.16041

**Published:** 2025-04-15

**Authors:** Parth Sushil Bajoria, Vinod Nookala

**Affiliations:** 1 GMERS Medical College and Hospital, Gandhinagar, India; 2 New York Medical College at St. Clare’s Health, Denville, NJ, USA

**Keywords:** Atrial fibrillation, dementia, digoxin immune Fab, digoxin toxicity, elderly patient.

## Abstract

Digoxin, a cardiac glycoside and sodium–potassium adenosine triphosphatase inhibitor, has a narrow therapeutic index and is primarily prescribed for conditions such as systolic heart failure and atrial fibrillation. This narrow window increases the risk of toxicity, especially among susceptible populations. Although digoxin use has declined in recent decades and cases of toxicity have become less frequent, clinicians must remain vigilant, particularly with geriatric patients, who are more susceptible due to polypharmacy and reduced renal function. Here, we present a case of a 77-year-old woman with dementia who exhibited elevated digoxin levels and was successfully treated with digoxin immune Fab. While the use of immune Fab in chronic toxicity cases remains uncertain, our retrospective review of similar cases, managed both with and without immune Fab, provides insights into its role and limitations. We further underscore the importance of regular digoxin monitoring rather than checking the levels only during toxic episodes, as consistent monitoring can prevent fatal cases and reduce overall mortality.

## Introduction

The use of digoxin has significantly decreased over the past two decades, and cases of toxicity have also become less common. However, providers must remain vigilant regarding various patient characteristics, such as age, muscle mass, kidney function, and concurrent use of other medications, to prevent potentially life-threatening toxicity.^1^

In this study, we report a case of digoxin toxicity in an elderly woman with dementia who presented with severe abdominal pain and vomiting and discuss the use of digoxin immune Fab in the management of such cases.

## Case presentation

A 77-year-old woman, a former smoker, with a medical history significant for dementia, psychosis, mood disorder, dysphagia status-post percutaneous endoscopic gastrostomy tube placement, and persistent atrial fibrillation presented to the emergency department with diaphoresis, multiple episodes of vomiting, abdominal pain, and distension. Vital signs showed a temperature of 102.1°F, heart rate of 171 beats/min, blood pressure of 168/72 mmHg, and respiratory rate of 29/min. An examination of systems revealed that the patient was confused and unable to follow commands, appeared severely dehydrated, had an irregular heart rhythm, and had diminished bowel sounds with a distended abdomen.

**Figure 1:**
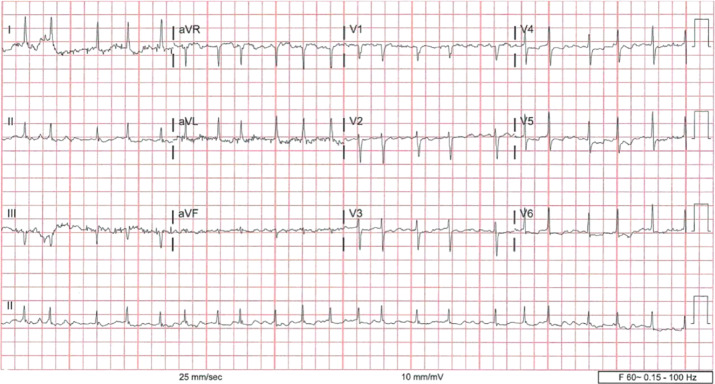
Electrocardiogram on admission suggestive of atrial fibrillation.

Her pupils were symmetric and measured 3 mm bilaterally, with the presence of both direct and indirect responses to light. No enlargement of the thyroid gland was detected. A 12-lead electrocardiogram (ECG) showed atrial fibrillation with a high ventricular rate, as depicted in **Figure 1**. Medications prior to admission included 2.5 mg of apixaban twice daily (BID), 125 μg of digoxin once daily (OD), 15 mg of mirtazapine OD, 20 mg of omeprazole suspension daily, and 250 mg of valproic acid daily.

Laboratory results were notable for leukocytosis with neutrophilia, high-normal potassium levels, mild hypercalcemia, a lactic acid level of 2.8 mmol/L, and a creatinine level of 0.82 mg/dL. Other significant laboratory values are noted in **Table 1**. She was empirically treated with intravenous (IV) ciprofloxacin and metronidazole, but urine and blood cultures and a chest X-ray ultimately came back negative, as did a test for severe acute respiratory syndrome antigen. A computed tomography scan of the abdomen and pelvis with contrast revealed severe rectal fecal impaction. Echocardiography indicated an ejection fraction of 45% with mild cardiomyopathy.

**Table 1:** Laboratory Results

Laboratory Values		
	Patient Value	Normal Value
WBC count	22,200/μL	4000–11,000/μL
Neutrophil count	19,300/μL	2500–7500/μL
RBC count	5.61 million/μL	3.5–5 million/μL
Hemoglobin	16.9 g/dL	11.7–13.8 g/dL
Hematocrit	49.6%	36%–46%
Potassium	4.7 mmol/L	3.5–5 mmol/L
BUN	19 mg/dL	7–20 mg/dL
Creatinine	0.82 mg/dL	0.6–1.2 mg/dL
Glomerular filtration rate	73.8 mL/min/1.73 m^2^	≥60 mL/min/1.73 m^2^
Lactate	2.8 mmol/L	0.5–2.2 mmol/L

*Abbreviations:* BUN, blood urea nitrogen; RBC, red blood cell; WBC, white blood cell.

After a comprehensive workup for sepsis, including cultures and imaging studies, yielded negative results, we were prompted to consider alternative explanations for the patient’s symptoms. Given her gastrointestinal complaints and ECG findings in the context of digoxin use, we measured her serum digoxin concentration, which ultimately confirmed toxicity. She was given IV fluids and digoxin immune Fab for acute reversal. Digoxin was discontinued, and metoprolol and diltiazem were given for rate control. The patient’s digoxin levels showed a rapid decrease after treatment with a total of six vials of 40 mg of digoxin immune Fab, and she showed clear symptomatic improvement within 24 h, as her bowel movements improved and her vitals normalized. In addition, the abdominal distention resolved, and her vomiting also subsided. Her laboratory results showed a downward trend in her digoxin concentration, which is presented in **Table 2**. Her leukocytosis resolved within 24 h, as did her lactic acidosis, which went down to 1.7 mmol/L. Her confusion gradually improved, and her mental status showed significant improvement, with a return to baseline cognitive function. She became alert, oriented, and was able to follow instructions appropriately, indicating the resolution of her neurological symptoms as the digoxin toxicity was corrected. On the seventh day of her admission, she was discharged to a skilled nursing facility in a hemodynamically stable condition.

**Table 2:** Trend of Blood Digoxin Level

Day of Admission	Blood Digoxin Level
Day 1	>10 ng/mL
Day 2	1.29 ng/mL
Day 4	0.16 ng/mL

Verbal consent was obtained from the patient for case publication.

## Discussion

Digoxin, a cardiac glycoside, has been used for more than two centuries for treating heart failure.^2^ It has also been used for over 50 years as a vital agent to control heart rate in patients with chronic atrial fibrillation, and, for more than 50 years, it has been a vital agent in controlling heart rate in patients with this condition.^3,4^ It exerts its therapeutic and toxic effects by blocking the sodium–potassium adenosine triphosphatase pump, which ultimately causes an increase in intracellular calcium in cardiac myocytes, which improves contractility. Additionally, it enhances the vagal tone, which leads to decreased conduction through the atrioventricular (AV) node. Digoxin’s therapeutic half-life is approximately 40 h, and kidneys are the main source for its elimination; therefore, elderly patients and those with chronic kidney disease are highly susceptible to toxicity with this medication. Digoxin toxicity has a high death rate, and its symptoms are very non-specific, which makes it difficult for providers to recognize it.^5,6^ The most frequent sign of digoxin toxicity is gastrointestinal disturbance, which occurs due to the direct stimulation of the chemoreceptor trigger zone in the medulla. Other rare presentations may be seen in clinical practice.^7^ Digoxin has numerous drug interactions, which, coupled with its narrow therapeutic index, often lead to toxicity, with patients presenting with a variety of symptoms and cardiac dysrhythmias. Toxic levels of this drug have also been associated with a greater risk of mortality in patients with heart failure or atrial fibrillation, even in the absence of overt toxicity symptoms.^8^

Here, we report a patient with a digoxin concentration of >10 ng/mL who was treated with digoxin immune Fab. The amount of this antidote to be delivered is calculated using the following formula:

$$\begin{array}{l} \text{Number of vials =}\\ \frac{\text{Serum digoxin concentration (ng / mL) }\times \text{ weight (kg)}}{100} \end{array}$$

This medication is indicated only for serious, life-threatening digoxin toxicity and not for cases with mild symptoms. Digoxin immune Fab works by binding to digoxin molecules in the blood and forming inactive complexes that are ultimately excreted by the kidneys. Indications to administer this drug include a serum digoxin level of 10 ng/mL (acute toxicity) or 6 ng/mL (chronic toxicity), end-organ dysfunction, fatal arrhythmia, cardiac arrest, or a serum potassium level of >5 mmol/L. Important limitations include high cost; limited shelf life; and adverse reactions such as heart failure exacerbations, hypokalemia, and allergic reactions.

Digoxin toxicity can either occur because of acute accidental over-ingestion or intentional overdosing or chronically because of drug accumulation over a long period of time. History plays a very important role for the clinician to diagnose digoxin toxicity; however, our patient presented with an altered mental status and was thus a poor historian. Whether the alteration in mental status was her baseline because of psychosis or was part of the clinical picture because of drug toxicity is hard to tell. Our patient presented with fever and an elevated blood count on admission and was treated with antibiotics.

Although the etiology for digoxin toxicity is not clear, our patient’s elderly age, polypharmacy, and baseline cognitive impairment clearly put her at an increased risk. Several drugs can also alter digoxin levels when prescribed concurrently, either by increasing or decreasing its blood concentration. An unusual cause of digoxin toxicity that we suspect in our patient is omeprazole. Kiley et al. described a 65-year-old woman on chronic digoxin therapy who developed toxicity a few months after starting omeprazole therapy.^7^ Other medications that raise its levels include metformin, antibiotics such as erythromycin and tetracyclines, antifungals, β-blockers such as carvedilol, calcium channel blockers, diuretics, rosuvastatin, angiotensin-converting enzyme inhibitors, anti-arrhythmics, angiotensin receptor blockers, and certain psychiatric medications.^6^ Punniyakotti and Begum reported a case of digoxin toxicity, likely secondary to concurrent diuretic use, and Xu and Rashkow reported a case of toxicity secondary to clarithromycin use. This highlights the importance of periodic monitoring of digoxin levels in patients taking multiple drugs to keep a check on drug–drug interactions and minimize the risk of toxicity.^9,10^

The most frequent sign of digoxin toxicity is gastrointestinal disturbance, although other rare presentations may be seen in clinical practice.^5^ Mannion et al. reported a rare case of digoxin toxicity in a 78-year-old woman who presented with intermittent diarrhea and vomiting.^11^ Their patient also developed chorea during her hospital stay secondary to drug toxicity. Elsewhere, Haruna et al. reported a case of digoxin toxicity in a patient with chronic heart failure manifesting as xanthopsia (yellow-tinted vision), which was due to the retinal toxicity of the drug.^12^

The most common electrocardiographic changes with digoxin toxicity include premature ventricular contractions, escape rhythms, or AV block. However, digoxin-induced increased myocardial activity can cause almost any type of cardiac arrhythmia.

Digiovanni-Kinsley et al. described a case of a 62-year-old male patient taking 125 μg of digoxin BID with a blood digoxin level of 2.65 ng/mL who was treated similarly to our case with digoxin immune Fab,^13^ while Bridwell et al. reported a case of a 75-year-old woman with systolic heart failure with a ventricular pacemaker in situ. Her digoxin levels were >10 ng/mL, similar to our case, but the authors did not use digoxin immune Fab to treat their patient, instead opting for supportive treatment. Their patient was discharged on Day 6 of her hospitalization in a stable condition.^14^ This highlights that the indications for the use of digoxin immune Fab are not consistent.

Rajpal et al. reported a case of a 53-year-old woman with acute digoxin toxicity due to an intentional overdose. Her blood digoxin level was 35.6 ng/mL. She was treated similarly with digoxin immune Fab, and her digoxin levels normalized with the resolution of atrial fibrillation. However, the next day, she developed recurrent arrhythmias, including ventricular fibrillation. Her subsequent digoxin level was 20.4 ng/mL, and the patient was treated with plasma exchange therapy.^15^ This report demonstrated plasma exchange therapy as a potential treatment for digoxin toxicity; however, further studies are required to prove its effectiveness.

## Conclusion

Our case addresses the challenges of identifying and managing digoxin toxicity in elderly patients with multiple comorbidities, especially an altered mental status, which makes it difficult to obtain patient history. Early recognition of symptoms such as gastrointestinal distress and altered mental status is critical for prompt diagnosis and treatment. We stress the importance of closely monitoring digoxin levels regularly in patients on chronic digoxin therapy who present with abdominal pain, as digoxin toxicity often manifests with non-specific symptoms. Regular assessment of blood levels is crucial for timely detection and management to prevent potential complications. With the rising prevalence of polypharmacy and drug interactions in the aging population, the current guidelines for digoxin monitoring need to be reformed, as studies have shown that higher doses of digoxin are linked to an increased risk of death. Relying solely on measuring digoxin levels in suspected cases of toxicity is no longer sufficient, and routine monitoring of drug levels is needed, especially for elderly patients, as the drug has a very narrow therapeutic index. The patient’s clinical improvement in our case was achieved through the use of digoxin immune Fab and supportive care. However, in chronic toxicity, the limited efficacy of this medication underscores the need for alternative treatments such as therapeutic plasma exchange, rifampicin, and dextrose–insulin infusions, although further research is needed to confirm their effectiveness.
